# Informational and structural barriers to exercise oncology care in Spain: development of the MOVE-Onco questionnaire and preliminary findings

**DOI:** 10.1007/s12094-026-04375-9

**Published:** 2026-05-18

**Authors:** Almudena Martínez Sánchez, Maria Mendoza-Muñoz, Francisco J. Dominguez-Muñoz, Candela Guerrero-Torrico, Narcis Gusi, Jeyanthi Subburaj, Santos Villafaina

**Affiliations:** 1 Grupo de Investigación en Actividad Física, Calidad de Vida y Salud (AFYCAV), Didáctica de la Expresión Musical, Plástica y Corporal, Facultad de Ciencias del Deporte, Universidad de Extremadura, Avenida de La Universidad S/N, 10003 Cáceres, Spain; 2 Instituto de Investigación e Innovación en Deporte (INIDE), 10003 Cáceres, Spain; 3 Department of Education and Sport, Universidad Loyola Andalucía, 41014 Seville, Spain; 4 Sri Venkateshwaraa College of Physiotherapy, Sri Venkateshwaraa Medical College Hospital and Research Centre, Puducherry University, 13-A, Villupuram Rd, Ariyur, Puducherry 605102 India

**Keywords:** Cancer survivorship, Exercise oncology, Social determinants of health, Healthcare accessibility, Supportive cancer care, Barriers and facilitators, Delphi technique

## Abstract

**Purpose:**

Exercise is recommended as a core component of supportive cancer care; however, its implementation remains inconsistent. Beyond individual motivation, social and healthcare system factors may shape access to structured exercise programs. This study aimed to develop and content-validate the Motivators, Obstacles, Values, and Exercise in Oncology (MOVE-Onco) questionnaire and to provide preliminary evidence on informational and structural barriers to exercise among oncology patients in Spain.

**Methods:**

A multi-phase study was conducted including: (1) qualitative item generation through patient interviews and cognitive debriefing; (2) a two-round Delphi process with a multidisciplinary expert panel (N = 16) using a ≥ 75% consensus criterion; and (3) a pilot cross-sectional application in 53 oncology patients to assess internal consistency and describe perceived barriers, facilitators, knowledge, and preferences.

**Results:**

Content validity was achieved with ≥ 75% expert agreement in the final Delphi round. Internal consistency was excellent for the Barriers scale (*α* = 0.91) and good for the Facilitators scale (*α* = 0.85). Although most participants believed exercise improves health outcomes (98.1%) and expressed interest in structured programs (86.9%), 67.9% reported not receiving professional exercise guidance and 94.3% were unaware of specialized oncology exercise resources. Informational barriers were more frequently endorsed than physical or psychological limitations in this pilot sample.

**Conclusions:**

The MOVE-Onco questionnaire demonstrates preliminary content validity and internal consistency in a Spanish oncology context. Pilot findings suggest that limited professional guidance and low awareness of available resources, rather than lack of patient motivation, may constitute key barriers to exercise engagement. These results highlight potential structural gaps in supportive cancer care delivery. However, these findings should be interpreted cautiously and require confirmation in larger studies.

**Supplementary Information:**

The online version contains supplementary material available at https://doi.org/10.1007/s12094-026-04375-9.

## Introduction

The paradigm of oncological care has undergone a profound transformation over the past two decades [1, 2]. Advances in early detection, precision medicine, and immunotherapies have substantially reduced cancer-related mortality, leading to a rapid expansion of the global population of cancer survivors [3, 4]. This epidemiological shift has compelled a transition from a predominantly acute, curative model of care toward a comprehensive survivorship framework that prioritizes long-term health outcomes and quality of life (QoL) [3, 5]. Within this evolving context, physical activity (PA) has emerged not merely as a general lifestyle recommendation, but as a powerful non-pharmacological adjuvant capable of attenuating treatment-related toxicities, reducing recurrence risk, and improving overall survival [6]. Accordingly, leading organizations such as the American College of Sports Medicine (ACSM) and the American Cancer Society (ACS) now explicitly recommend that cancer survivors avoid physical inactivity and accumulate 150–300 min of moderate-intensity PA per week, reflecting a paradigm shift in which exercise is increasingly recognized as a core component of standard oncological care rather than an optional wellness strategy [7, 8].

Despite the robust and growing epidemiological and clinical evidence supporting the benefits of PA in cancer survivorship, its integration into survivors’ daily lives remains markedly suboptimal. Surveillance data indicate that approximately 36.7% of cancer survivors report no engagement in leisure-time physical activity [9], while only around 4% fully comply with the combined nutrition and physical activity guidelines issued by major health organizations [10]. Although some studies suggest those who meet at least one exercise component achieve adherence up to 72%, combined exercise (aerobic + resistance) falls below 20% [11]. Accumulating evidence indicates that exercise participation and maintenance in cancer survivors are shaped by a complex and dynamic interplay of demographic, clinical, psychological, physical, social, and environmental factors [12].

Despite strong clinical recommendations, access to structured exercise programs is not uniformly integrated into routine oncology care. Emerging evidence suggests that beyond individual-level factors, healthcare-system characteristics, availability of specialized services, referral pathways, geographic distribution of resources, and health literacy may substantially influence exercise engagement [13]. These elements are closely linked to the broader framework of social determinants of health, which shape disparities in access to supportive cancer care. In this context, exercise participation may reflect not only personal motivation or physical capacity, but also structural and informational inequities within healthcare systems [14].

Qualitative and quantitative evidence consistently identifies a range of modifiable barriers such as treatment-related side effects, lack of knowledge about exercise benefits and safety, motivational deficits, and logistical constraints [15]. Conversely, several facilitating factors have been shown to promote engagement in physical activity, notably perceived social support, guidance and encouragement from healthcare professionals, and the alignment of exercise interventions with individual preferences in terms of modality and setting [15]. There are existing measurement tools that focus on quantifying activity volume such as the Godin–Shephard Leisure-Time Physical Activity Questionnaire (GSLTPAQ) [16] and the International Physical Activity Questionnaire (IPAQ) [17]. However, these instruments provide limited insight into the behavioral mechanisms underlying physical activity engagement. This methodological limitation substantially constrains their usefulness for informing the design of tailored, patient-centered exercise interventions, underscoring the need for measurement tools that move beyond “how much” physical activity is performed to elucidate “why” survivors do or do not engage in exercise.

In this context, the recent questionnaire developed by Subburaj and Sharma (2023) [18] represents a significant methodological advancement. Unlike previously available instruments, this questionnaire was specifically designed to examine the multidimensional determinants of exercise behavior within cancer survivorship. This instrument systematically addresses four key domains: (1) knowledge, encompassing understanding of exercise safety, benefits, and guidelines during and after cancer treatment; (2) barriers, including factors that hinder participation such as fatigue, nausea, fear of injury, or lack of time; (3) facilitators, referring to elements that promote engagement, such as healthcare professional recommendations or social support; and (4) preferences, covering desired exercise modalities, intensity, and timing. While conceptually valuable, this questionnaire does not include a Likert-scale format, limiting its ability to quantify the extent or strength of patient barriers, and facilitators. These limitations, together with the need for cultural adaptation and the inclusion of additional context-specific domains, motivated the development of a new instrument designed to be both culturally appropriate and quantifiable, enabling more precise assessment and comparison of patient responses.

The development of patient-reported instruments in oncology requires methodological approaches grounded in established evidence and previous research. Prior studies on questionnaire development in health sciences have consistently recommended multi-phase processes that integrate qualitative item generation, patient involvement, and structured expert consensus to ensure conceptual relevance and clinical applicability [19, 20]. Following these methodological models, often used in the creation of physical activity and supportive-care instruments for cancer populations, the present study adopts a sequential, evidence-based design to develop the Motivators, Obstacles, Values, and Exercise in Oncology (MOVE-Onco) questionnaire. This approach builds directly on earlier work demonstrating the value of open-ended question elicitation, cognitive interviewing, and Delphi consensus for establishing content validity and refining instrument structure.

In light of the need to better understand not only individual motivations but also potential structural and informational barriers influencing exercise engagement in oncology care, the objective of this study was twofold: (1) to develop and content-validate the Motivators, Obstacles, Values, and Exercise in Oncology (MOVE-Onco) questionnaire using a structured Delphi process; and (2) to provide preliminary descriptive evidence on perceived knowledge gaps, barriers, facilitators, and exercise preferences among oncology patients in Spain. By identifying patient-level and system-level determinants, this instrument aims to contribute to the assessment of supportive care accessibility within the Spanish oncology context.

## Methods

**Fig. 1:**
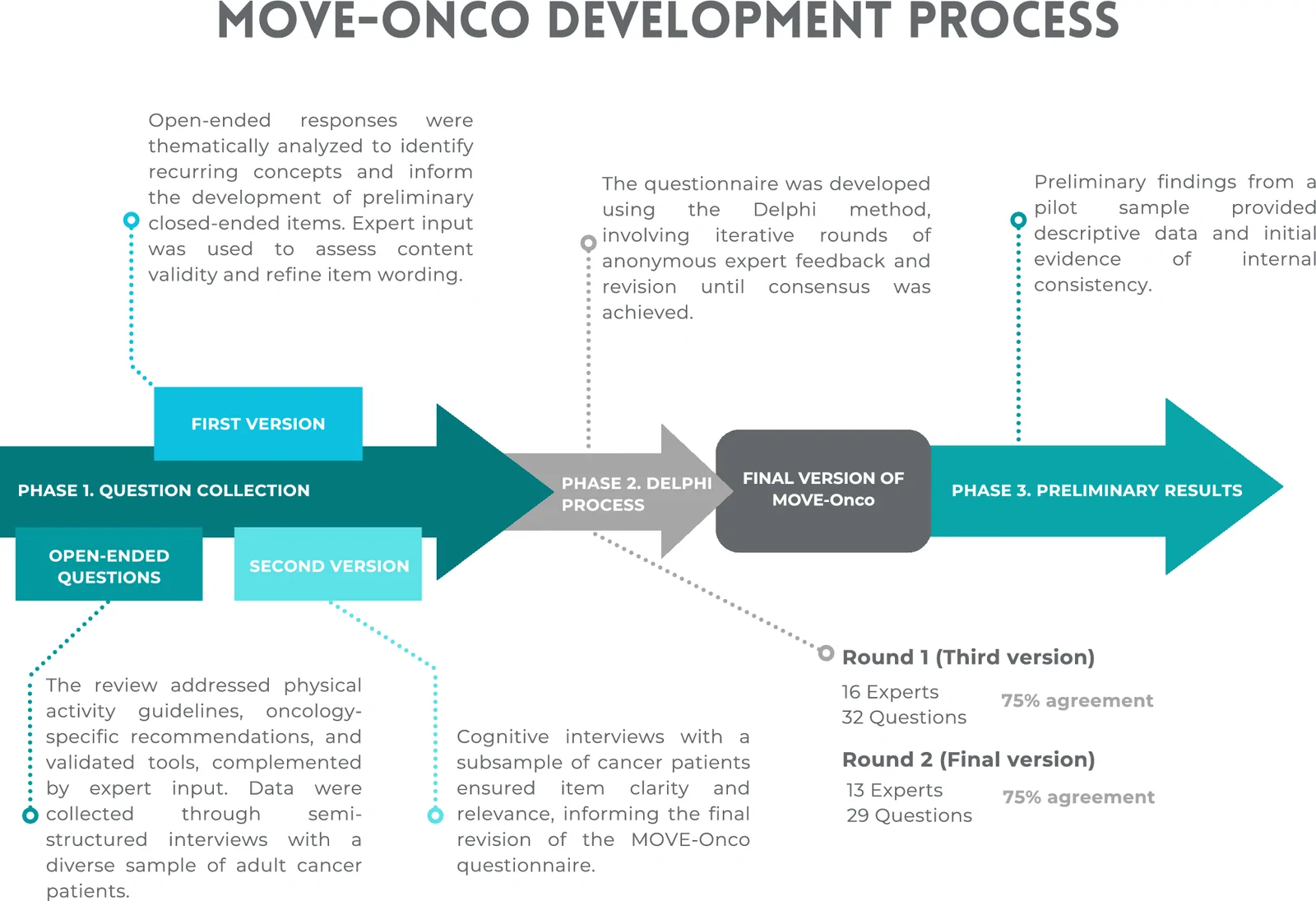
Motivators, obstacles, values, and exercise in Oncology (MOVE-Onco) questionnaire development process

This research follows a structured multi-phase process consistent with established methodological standards for health measurement instrument development [19–21]. A sequential design was implemented, combining qualitative procedures for item generation with expert-based content validation and preliminary psychometric assessment. The process comprised three phases based on previous studies: 1) question collection (item development through open-ended questions and cognitive interviews) [19, 20]; 2) expert consensus using a Delphi technique to assess item relevance, clarity, and representativeness [21–23]; and 3) preliminary quantitative analysis focused exclusively on internal consistency reliability of the instrument. All these phases are summarized in Fig. 1.

All study procedures were conducted in accordance with the Declaration of Helsinki and approved by the University of Extremadura Ethics Committee (Approval No. 39/2025).

### Phase 1. Question collection

#### Open-ended questions

The thematic structure of the Motivators, Obstacles, Values, and Exercise in Oncology (MOVE-Onco) questionnaire was established through a comprehensive literature review focused on physical activity guidelines and recommendations specific to oncology populations [6, 24, 25], facilitators and barriers to physical exercise in oncology populations [15], as well as validated tools used in similar clinical settings [18].

Initial draft items were generated in open-ended format to ensure that the questionnaire reflected real experiences, language, and perspectives of oncology patients (Supplementary Material 1). This version was generated by an expert group of medical doctors, nurses, and exercise professionals. These open responses were gathered through semi-structured interviews conducted with a convenience sample of adult cancer patients recruited from oncology-related clinical and community settings in Spain, including hospitals and patient associations. Eligible participants were required to be ≥ 18 years of age, have a current or previous cancer diagnosis, and be able to understand and respond to the interview questions. Patients with cognitive impairment or any condition that prevented effective communication were excluded.

#### First version of MOVE-Onco

Responses to the open-ended questions were then analyzed thematically to identify recurring concepts and phrasing, which were used to create preliminary closed-ended items (First version; Supplementary Material 2). Expert feedback was subsequently gathered from medical doctors, nurses, and exercise professionals to assess content validity and refine question wording.

#### Second version of MOVE-Onco

The information obtained from these interviews was used to ensure the clarity, relevance, and appropriateness of the items. This feedback informed the revision and finalization of the second version of the MOVE-Onco questionnaire (Supplementary Material 2).

### Phase 2. e-Delphi process

An electronic Delphi (e-Delphi) technique was used to obtain structured, iterative expert consensus on the relevance and clarity of the MOVE-Onco items [26]. The e-Delphi approach enables anonymous participation and remote completion, reducing dominance bias and facilitating multidisciplinary input without face-to-face meetings [27]. Two rounds were conducted in January 2025, with the second round completed by January 31st. Google Forms was used, and all communication was performed by email.

Eligible experts were oncologists (≥ 3 years of clinical experience), oncology nurses (≥ 3 years of recent experience), exercise professionals/scientists specialized in cancer survivorship, and health researchers (a minimum master’s degree, preferably with expertise in questionnaire development). Experts were contacted by email and subsequently provided with the draft questionnaire accompanied by a cover letter with the e-Delphi methodology and instructions for completion (see Supplementary Material 4).

*Round I*. Experts rated each item for relevance (yes/no) and clarity (clear/unclear) and provided open-ended suggestions for wording and response options. Items reaching the a priori consensus threshold (≥ 75% agreement on relevance) were retained; items below the threshold were removed or revised based on qualitative feedback.

*Controlled feedback and iteration*. Between rounds, the research team synthesized quantitative results (agreement percentages) and qualitatively summarized recurring comments and proposed edits. Revised items were incorporated into the subsequent round. The feedback provided to the panel was aggregated and anonymized. This resulted in the third version of the questionnaire (Supplementary Material 2).

*Round II*. Experts re-evaluated the revised item set and rated each item’s relevance on a 5-point Likert scale (1 = not relevant to 5 = highly relevant). Ratings of 4–5 were considered endorsements, with consensus defined as ≥ 75% endorsement. Experts could additionally comment on wording and comprehensibility.

*Attrition*. Experts who did not respond within the round’s deadline were not included in subsequent rounds; response rates per round are reported in the Results section.

### Phase 3. Descriptive preliminary findings

To obtain preliminary descriptive data and assess internal consistency, the final version of the MOVE-Onco questionnaire was administered in a pilot cross-sectional sample of oncology patients (*n* = 53).

Participants were recruited through convenience sampling from oncology-related clinical and community settings in Spain, including hospitals and patient associations. Eligibility criteria included: (i) being ≥ 18 years of age, (ii) having a current or previous cancer diagnosis, and (iii) being able to understand and complete the questionnaire independently. All participants received detailed information about the study procedures and potential risks and provided written informed consent prior to participation. The research protocol was approved by the Bioethics and Biosafety Committee of the University of Extremadura (approval reference number: 39/2025) and was conducted in accordance with the Declaration of Helsinki.

Participants completed a structured self-report questionnaire to collect sociodemographic and clinical information, including sex, age, educational level, and clinical status related to the oncological process. In addition, physical activity levels were assessed using the Godin Leisure-Time Exercise Questionnaire, which was used to categorize participants according to their level of physical activity [16].

### Statistical analysis

In each Delphi round, expert agreement on the relevance of the items was assessed by calculating proportions and mean scores. A consensus cut-off of 75% was set for the first round [28, 29]. In the second round, experts rated each item on a 5-point scale (1 = the item does not fit the questionnaire; 5 = the item fits the questionnaire), and ratings of 4 or 5 were considered agreement. The consensus threshold for this round was set at 75%. Prior to psychometric analysis, two items (from the facilitators section) were reverse-coded so that higher Likert-scale scores uniformly represented greater endorsement of the underlying construction. Internal consistency reliability was subsequently assessed by calculating Cronbach’s alpha coefficients for the barriers’ and facilitators’ subscales. Cronbach’s alpha was selected as a widely accepted measure of internal consistency to determine the extent to which items within each domain are interrelated and reliably measure the same underlying construct. Alpha values ≥ 0.70 are generally considered acceptable, values between 0.80 and 0.90 indicate good reliability, and values above 0.90 are considered excellent [30]. All analyses were conducted using SPSS (IBM SPSS Statistics, version 27.0; Armonk, NY: IBM Corp.). Given the exploratory nature and sample size of this pilot study, no analyses of factor structure, construct validity, or test–retest reliability were performed.

## Results

### Phase 1. Question collection

The thematic structure of the MOVE-Onco was defined through a comprehensive review of the literature on physical activity guidelines and recommendations for oncology populations [6, 15, 24, 25], as well as previously validated instruments used in similar clinical settings [18]. This process allowed the identification of the key domains relevant to physical activity behavior among individuals undergoing or recovering from cancer treatment. Based on this analysis, a set of core content areas was identified to reflect the most relevant aspects of physical activity engagement in individuals undergoing or recovering from cancer treatment. These content areas include:

1. General questions: general questions about physical exercise routines, aspects related to travel and caregivers in the event of illness.
2. Preferences: interest in exercise programs, type of physical exercise, issues related to the time spent on physical exercise, where to do it, with whom to do it, who they would prefer to lead it, when they would like to start, and how they would like to receive information about these programs.
3. Knowledge: about the adverse effects of physical exercise, its benefits, when they should start an exercise program, and whether anyone has taught them anything about it.
4. Barriers: reasons for not participating in physical exercise programs, accessibility, and physical factors.
5. Facilitators: reasons and people who motivate them to do physical exercise and people with whom they would be motivated to do it.

Initial items were developed in an open-ended format to capture the authentic experiences, language, and perspectives of oncology patients (Supplementary Material 1). These preliminary questions were created collaboratively by an expert group consisting of oncologists, nurses, and exercise professionals and were administered through semi-structured interviews in a heterogeneous sample of adult cancer patients at different stages of treatment and recovery.

Responses to the open-ended questions were subjected to thematic analysis in order to identify recurring concepts, keywords, and patterns in participants’ narratives. These emerging themes were subsequently transformed into preliminary closed-ended items, resulting in the first version of the questionnaire (Supplementary Material 2).

Subsequently, cognitive interviews were conducted with a subsample of 18 cancer patients (age *M* = 59.1, *SD* = 13.3; time since diagnosis *M* = 6.2, *SD* = 7.4) recruited from oncology-related clinical and community settings in Spain. The subsample included participants with different educational levels (compulsory education, high school, vocational training, and university), a variety of cancer types (including breast, colon, colorectal, retroperitoneal, Hodgkin lymphoma, melanoma, leukemia, ovarian, cervical, and prostate cancers), and different clinical stages (*n* = 8 undergoing active treatment; *n* = 10 in remission), ensuring heterogeneous perspectives during item refinement. These cognitive interviews were used to assess item clarity, comprehension, and relevance, and to revise and finalize the second version of the MOVE-Onco questionnaire (Supplementary Material 2). Following these cognitive interviews, the questionnaire underwent a series of systematic refinements aimed at improving clarity, expanding response options, and optimizing item wording. In the general section, the item regarding participation in structured exercise programs was revised, and only the item assessing regular engagement in physical exercise was retained. More precise response formats were introduced to quantify the frequency and duration of exercise prior to cancer diagnosis. Several transportation-related response options were modified; for instance, “rental car” was replaced with “taxi” and “bicycle” was added. In the Preferences section, the range of exercise modalities was substantially broadened to include both individual and team-based sports, horseback riding, and a clearer specification of activities such as Yoga/Pilates and supervised training. The wording of multiple items was refined to enhance comprehensibility (e.g., “Is it feasible for you to make time…?”). Furthermore, in the Knowledge section, the list of potential benefits of physical exercise was expanded to incorporate psychological and social dimensions, including reductions in anxiety and depression, and improvements in social participation. In the Barriers and Facilitators sections, the use of Likert-type response scales was formally structured, and additional items addressing lack of support, motivational factors, and financial and accessibility barriers were incorporated.

### Phase 2. Delphi process

The expert panel comprised professionals from oncology medicine, oncology nursing, exercise science, and health research, ensuring multidisciplinary representation. Sixteen experts were invited and completed Round 1, and thirteen completed Round 2. All participants were from Spain.

Of the 32 items, 29 exceeded the 75% consensus threshold. Only three items did not reach the 75% expert agreement threshold and were therefore removed for the subsequent round. The agreement percentages for each item are presented in Supplementary Material 3.

In addition, several expert suggestions were incorporated to refine specific items (See Supplementary Material 2). For example, in the question *“Who takes care of you when you are ill?”* the response option *“Personal assistant/home caregiver”* was added. In the question *“Who would you like to lead the physical exercise program?”* the response options *“Myself, using information obtained from the Internet”* and *“Myself, using information obtained from books and physical exercise guidelines”* were included. Within the barriers section, the following response options were added: *“Health professionals have not informed me about how to correctly perform physical exercise given my diagnosis”* and *“I have domestic responsibilities that prevent me from doing so”.* Finally, for both the barriers and facilitators sections, one of the most recurrent suggestions from the panel was to clarify the evaluation scale; therefore, the following statement was added: *“using a scale from 1 to 5, where 1* = *Completely disagree and 5* = *Completely agree.”*

In the second round, 29 items were included in the questionnaire (see Supplementary Material 2). The 16 professionals who responded in the first round were invited to participate again and received a link to the online survey, of whom 13 completed it. Expert representation from all specialties was maintained in this round. A score of 4 or higher on a 5-point Likert scale was considered indicative of relevance. All items were deemed relevant and achieved the predefined 75% agreement threshold. The specific agreement percentages for each item are presented in Supplementary Material 3. Most of the comments received from the panel during the second round were related to wording rather than content, and the corresponding revisions were made.

### Phase 3. Descriptive preliminary findings

Table 1 outlines the sociodemographic and clinical profile of the study sample (N = 53). The participants had a mean age of 54.38 years and were predominantly female (n = 45). Regarding educational attainment, the largest proportion of the sample held a university degree or postgraduate qualification (43.2%), followed by high school education (33.3%) and compulsory education (23.5%).

**Table 1:** Sociodemographic and clinical characteristics of the study sample (*n* = 53)

Variables	Category	Percentage (%)/Mean (SD)
Gender	Male	8 (15%)
	Female	45 (85%)
Age (years)		54.38 (9.43)
Educational level	Compulsory education	23.5%
	High School	33.3%
	University degree/Postgraduate	43.2%
Clinical status	Active treatment	38 (71.7%)
	Survivor (post-treatment)	15 (28.3%)
Physical activity level	Active	62.3%
	Moderately active	18.9%
	Insufficiently active	18.9%

*SD* standard deviation

In terms of clinical status, the majority of participants were undergoing active treatment (71.7%), while 28.3% were classified as cancer survivors (post-treatment). Breast cancer was the most prevalent diagnosis (71.7%), followed by colon cancer (7.5%) and lymphoma (3.8%), with other typologies (e.g., ovarian, prostate, thyroid) appearing with lower frequency (1.9% each).

Regarding baseline physical activity levels, a substantial portion of the sample was classified as active (62.3%). The remaining participants were equally distributed between the moderately active (18.9%) and insufficiently active (18.9%) categories.

Table 2 details participants’ preferences concerning the structure and supervision of physical exercise programs. The vast majority of participants expressed a high willingness to engage in physical activity, with 86.9% explicitly stating interest in participating in an exercise program. Regarding specific modalities, walking was the most preferred activity (75%), followed by supervised personal training (64.2%) and mind–body activities such as Yoga or Pilates (64%).

**Table 2:** Patients’ preferences regarding physical exercise

Section/Question	Response	Percentage (%)
Ability to allocate time	Yes	67.9%
	No	11.3%
	Sometimes	20.8%
Interest in participation	Yes	86.9%
	No	11.3%
	I am not sure	0.0%
Preferred exercise modalities (Top 5 selected)	Walk	75.0%
	Personal training	64.2%
	Yoga/Pilates	64.0%
	Flexibility exercises	60.4%
	Lift training	52.8%
Perceived sustainable duration	< 10 min	0.0%
	10 – 20 min	9.4%
	20 – 30 min	28.3%
	> 30 min	62.3%
Preferred timing	Morning	54.7%
	Mid-afternoon	22.6%
	Late afternoon/Evening	9.4%
	No preference	20.8%
Preferred place	Home	20.8%
	Gym/Fitness center	54.7%
	Park/outdoors	28.3%
	Patient association	39.6%
	Multidisciplinary center	34%
	Hospital / health center	3.8%
Preferred company	Alone (with personal trainer)	37.7%
	With other cancer survivors	60.4%
	General public	49.1%
	With family members	13.2%
	Friends	22.6%
Preferred supervisor	Physiotherapist	45.3%
	Oncologist	9.4%
	Personal trainer	83%
	Self-directed (internet)	5.7%
	Self-directed (book)	1.9%
Preferred timing to start the program	Before treatment	35.8%
	During treatment	30.2%
	0–6 months post-treatment	17%
	7–12 months post-treatment	7.5%
	> 1-year post-treatment	9.4%
Preferred means to receive information regarding physical exercise programs (Top 3 selected)	Informative talk	69.8%
	App with exercises	34%
	Videos	20.8%

In terms of logistical preferences, participants predominantly favored sessions lasting more than 30 min (62.3%) and conducted in the morning (54.7%). Gyms or fitness centers (54.7%) and patient associations (39.6%) were identified as the preferred settings. Notably, supervision was deemed essential; a significant majority (83%) indicated a preference for programs led by a personal trainer, whereas self-directed exercise using the internet (5.7%) or book resources (1.9%) received minimal support. Socially, participants expressed a preference for exercising with other cancer survivors (60.4%) or the general public (49.1%), rather than alone.

Table 3 summarizes the participants’ knowledge and beliefs regarding exercise during and after cancer treatment. While belief in the general health benefits of exercise was nearly universal (98.1%), a significant gap in professional guidance was identified. Approximately 67.9% of respondents reported that they had not received specific instruction from healthcare professionals regarding how to exercise post-diagnosis.

**Table 3:** Patients’ knowledge regarding physical exercise

Section/Question	Response	Percentage (%)
Knowledge of adverse effects (Top 3 selected)	No adverse effects	49.1%
	Injuries	26.4%
	Fatigue	24.5%
Knowledge of benefits (Top 3 selected)	Improve strength	94.3%
	Improve or maintain health	92.5%
	More socially active	88.7%
Received professional instruction	Yes	32.1%
	No	67.9%
	Yes, but I do not remember the exercises	0.0%
Optimal timing to start	During treatment	73.6%
	0–3 months post-treatment	9.4%
	3–6 months post-treatment	9.4%
	7–12 months post-treatment	5.7%
	> 1-year post-treatment	1.9%
Belief in health benefits	Yes	98.1%
	No	1.9%
	Maybe	0.0%
Awareness of local resources	Yes	5.7%
	No	94.3%

Furthermore, misconceptions regarding safety were observed; nearly half of the sample (49.1%) believed that exercise entails "no adverse effects," suggesting a potential lack of awareness regarding specific contraindications or necessary precautions. Despite this, the majority correctly identified improvements in strength (94.3%) and overall health maintenance (92.5%) as key benefits. However, knowledge of local resources remains a critical barrier, with 94.3% of participants indicating they were unaware of specialized oncology exercise centers.

Table 4 presents the descriptive statistics and internal consistency for the perceived barriers to physical exercise. The overall internal consistency of the barriers section was excellent (α = 0.911), with subscales demonstrating acceptable to good reliability: General Barriers (α = 0.807), Barriers Despite Knowledge (α = 0.795), and Access/Health Barriers (α = 0.849).

**Table 4:** Internal consistency and descriptive statistics of the perceived barriers

Subscale/item description	Mean (SD)	Cronbach´s α
I. Perceived barriers		0.911
B1. General barriers (Question 1)		0.807
a. I feel too tired to exercise	2.19 (1.33)	
b. I do not have enough strength	2.23 (1.30)	
c. I do not believe exercise makes a difference in my health	1.38 (1.01)	
d. Pain prevents me from exercising	1.98 (1.15)	
e. I am afraid of falling	1.42 (0.93)	
f. I feel shortness of breath / difficulty breathing	1.67 (1.14)	
g. Professionals have not informed me about benefits	2.64 (1.57)	
h. Professionals have not taught me how to exercise correctly	3.28 (1.68)	
i. I do not have time	1.40 (1.01)	
j. I lack motivation or desire	2.08 (1.43)	
k. I cannot afford it financially	1.85 (1.21)	
l. I do not know of specialized oncology exercise centers	3.64 (1.77)	
m. I feel a lack of support from my environment	1.53 (1.22)	
n. I feel embarrassed/ashamed	1.40 (0.91)	
o. Domestic responsibilities prevent me	1.81 (1.30)	
B2. Barriers despite knowledge/recommendation (Question 2)		0.795
a. I cannot get started with exercise	2.13 (1.43)	
b. I am unable to maintain an exercise routine	2.38 (1.51)	
c. The price of exercise services is high	2.56 (1.61)	
d. Progress in treatment effectiveness is slow	2.28 (1.35)	
e. I do not feel safe going out to exercise	1.60 (1.04)	
B3. Access and health condition barriers (Question 3)		0.849
a. Overweight reduces my participation	1.60 (1.18)	
b. Pain reduces my participation	2.04 (1.36)	
c. Swelling or lymphedema prevents me	1.91 (1.35)	
d. Joint stiffness reduces my participation	2.19 (1.41)	
e. Urinary incontinence issues reduce my participation	1.49 (0.93)	
f. No one supports me to exercise	1.43 (1.03)	
g. I feel embarrassed when exercising	1.45 (0.99)	
h. I have no mood/spirit to exercise	2.08 (1.36)	
i. Family responsibilities reduce my participation	1.92 (1.43)	

Responses were rated on a 5-point Likert scale, where 1 = strongly disagree and 5 = strongly agree. Two items were reverse-coded

*SD* standard deviation

Results indicate that informational barriers were more prevalent than physical or psychological ones. The highest-rated barrier was the lack of knowledge regarding resources; specifically, the item *"I do not know of specialized oncology exercise centers"* obtained the highest mean score (*M* = 3.64, *SD* = 1.77). This was followed by the perception that *"Professionals have not taught me how to exercise correctly"* (*M* = 3.28, *SD* = 1.68) and *"Professionals have not informed me about benefits"* (*M* = 2.64, *SD* = 1.57).

Conversely, intrapersonal and social barriers were among the least endorsed. Participants reported low levels of embarrassment (*M* = 1.40, *SD* = 0.91) and generally felt supported by their environment (*M* = 1.53, *SD* = 1.22). Among the physical barriers, joint stiffness (*M* = 2.19, *SD* = 1.41) was the most notable limiting factor, though its overall impact was moderate compared to the lack of specialized information.

Table 5 outlines the facilitators and motivators for engaging in physical exercise, showing good internal consistency for the global scale (*α* = 0.849) and its subscales, particularly Social Motivation (*α* = 0.919). Motivational Factors (F1 and F5) emerged as strong drivers for participation. The desire to prevent relapses was the highest-rated motivator in the entire questionnaire (*M* = 4.81, *SD* = 0.68), followed closely by the aim to improve quality of life (*M* = 4.66, *SD* = 0.88) and the intrinsic factor *“If I enjoyed doing it”* (*M* = 4.62, *SD* = 0.84). Regarding Social Motivation (F2), friends (M = 4.06, SD = 1.27) and relatives (*M* = 3.98, *SD* = 1.31) were identified as slightly stronger sources of motivation compared to partners (*M* = 3.89, *SD* = 1.51) or siblings (M = 3.78, SD = 1.43). Finally, conditions for participation (F4) highlighted the importance of accessibility. Participants strongly agreed that they would join a program if the center were *“nearby”* (M = 4.34, SD = 1.13) and if the schedule were flexible (M = 4.28, SD = 1.21). Cost was also a relevant factor (M = 4.13, SD = 1.40), reinforcing the need for accessible programming.

**Table 5:** Internal consistency and descriptive statistics of the perceived facilitators

Subscale/Item description	Mean (SD)	Cronbach´s α
II. Perceived facilitators (Sect. “Discussion”)		0.849
F1. Motivational factors (Question 1)		0.864
a. Help maintain balance, strength, and mental acuity	4.45 (1.07)	
b. Feeling renewed/refreshed after exercise	4.42 (1.17)	
c. Preventing deterioration of my current state	4.58 (0.93)	
d. Improving quality of life	4.66 (0.88)	
e. Knowing the benefits beforehand	4.25 (1.11)	
F2. Social motivation (Source) (Question 2)		0.919
a. Partner motivates me	3.89 (1.51)	
b. Siblings motivate me	3.78 (1.43)	
c. Children motivate me	3.90 (1.43)	
d. Friends motivate me	4.06 (1.27)	
e. Relatives motivate me	3.98 (1.31)	
f. No one motivates me (Reverse scored if applicable)	1.56 (1.11)	
F3. Company/accompaniment (Question 3)		0.825
a. If my partner accompanied me	3.45 (1.39)	
b. If my siblings accompanied me	2.78 (1.42)	
c. If my children accompanied me	3.47 (1.43)	
d. If a friend accompanied me	3.69 (1.50)	
e. If a relative accompanied me	3.20 (1.50)	
f. If no one accompanied me	3.02 (1.54)	
g. If my pet accompanied me	2.47 (1.46)	
F4. Conditions for participation (Question 4)		0.780
a. If it were low cost or free	4.13 (1.40)	
b. If the center were nearby	4.34 (1.13)	
c. If someone accompanied me	3.08 (1.54)	
d. If it were accessible (transport/parking)	3.92 (1.30)	
e. If sessions were in a group with other patients	4.04 (1.11)	
f. If the schedule were flexible	4.28 (1.21)	
F5. Regular practice motivators (Question 5)		0.803
a. If I enjoyed doing it	4.62 (0.84)	
b. If my family motivated me	3.60 (1.50)	
c. If it prevented relapse	4.81 (0.68)	
d. If I felt I had control over my health	4.42 (1.12)	
e. If it reduced treatment side effects	4.57 (1.05)	

Responses were rated on a 5-point Likert scale, where 1 = strongly disagree and 5 = strongly agree. Two items were reverse-coded

*SD* standard deviation

## Discussion

This study developed and preliminarily evaluated the MOVE-Onco questionnaire, a patient-centered instrument designed to assess knowledge, barriers, facilitators, and preferences related to exercise in oncology. Beyond its methodological contribution, the pilot findings reveal an important pattern: despite high levels of motivation and strong belief in the benefits of exercise, most participants reported limited professional guidance and low awareness of specialized exercise resources. These findings suggest that, in this sample, informational factors—and potentially structural aspects—may be relevant in shaping exercise engagement. However, given the descriptive and cross-sectional nature of the data, these results should be interpreted cautiously and cannot establish causal relationships or confirm system-level deficiencies. From an equity perspective, these findings may be compatible with the hypothesis that exercise engagement is influenced by social and healthcare system determinants, including access to reliable information, availability of specialized services, and the existence of referral pathways [14]. In settings where exercise oncology is not systematically integrated into routine care, patients’ opportunities to participate could be influenced by territorial availability of resources and the way information is delivered within healthcare services.

The relevance of this contribution is underscored by the limited availability of validated, patient-centered instruments in Spanish tailored to the oncology context. MOVE-Onco was informed by current physical activity guidelines for oncology populations and evidence on barriers and facilitators to exercise participation [6, 15, 24, 25], as well as by previously validated multidimensional instruments developed in similar settings [18]. Although Subburaj and Sharma [18] proposed a questionnaire addressing comparable domains, direct application without cultural and contextual adaptation may compromise validity. While retaining its modular structure, MOVE-Onco introduces key improvements: (i) the use of 5-point Likert scales to quantify the intensity of perceived barriers and facilitators, enabling internal consistency assessment in line with established psychometric standards [30]; and (ii) the explicit inclusion of system-level barriers, such as lack of professional guidance and limited awareness of exercise resources, not previously captured [18]. These additions proved essential, as informational deficits emerged as more prominent barriers than fatigue in this sample.

A major strength of MOVE-Onco is its rigorous, multi-phase development process combining patient input and multidisciplinary expert consensus. This approach follows established recommendations for questionnaire development that emphasize patient-informed item generation and expert refinement to ensure conceptual and contextual adequacy [19, 20]. Cognitive interviews enhanced clarity and acceptability, and the two-round Delphi process provided structured content validation using a predefined consensus threshold [28, 29]. Importantly, expert feedback reinforced the inclusion of clinically relevant but often underrepresented barriers, such as lack of professional guidance and domestic responsibilities, supporting the content validity and conceptual coherence of the instrument.

Preliminary findings of the application of MOVE-Onco to a sample of cancer patients showed a disconnect between patients’ motivation for exercise and actual healthcare support. Although nearly all participants acknowledged exercise benefits (98.1%) and a large majority expressed interest in structured programs (86.9%), most reported no professional guidance on how to exercise (67.9%), and almost all were unaware of specialized centers (94.3%). While classic barriers in cancer populations have often emphasized physical limitations or treatment-related fatigue [15], contemporary evidence increasingly points to organizational and informational gaps as key impediments [13]. Previous literature suggests that healthcare systems may lack formal structures for exercise integration, referral pathways, and resources to support tailored programs, which could limit patients’ ability to act on their motivation. Studies synthesize multiple barriers at organizational and clinician levels, including insufficient time, unclear referral processes, and limited clinician training in exercise oncology communication [13]. Moreover, the low scores for physical and psychological barriers in our sample (contrasted with high scores for informational barriers) suggest that, in this cohort, informational and structural factors may play a more prominent role than motivational deficits. Even among well-educated participants, specific literacy about exercise in cancer care appears insufficient, aligning with reports that clinicians can struggle to operationalize exercise recommendations due to uncertainty about how to counsel or refer patients effectively [31, 32]. In this regard, a previous study showed that only 18.3% of specialists referred patients to an exercise program [32]. Despite the recognition of exercise’s role in oncology among patients and clinicians, healthcare systems often lack formal structures, clear referral pathways, and resources to support individualized programs. When supervised programs are not embedded within public healthcare systems, access may depend on personal financial resources or geographic proximity, which may contribute to disparities in supportive cancer care [14]. Moreover, healthcare professionals themselves report significant barriers, such as limited consultation time and insufficient specific knowledge about exercise-oncology guidelines, resulting in vague advice like “walk more” instead of structured prescriptions [33, 34].

Moreover, preliminary findings may help health professionals with how exercise interventions should be structured. Nearly half of participants (49.1%) believed that exercise carries no risk of adverse effects, which, alongside a strong interest in strength training (52.8%), reveal potential for engagement in activities that could pose injury risks without appropriate supervision. Consistent with this, 83% of participants preferred programs led by an exercise professional, with minimal interest in self-directed exercise through online resources or books (< 6%). This aligns with literature showing that cancer survivors value structured, supervised programs that provide tailored guidance and reassurance of safety (preferences often linked to greater adherence and confidence in exercise participation) [35, 36]. Additionally, a majority favored group exercise settings (60.4%), underscoring the relevance of social support in motivation and engagement. Previous literature supports the idea that social support is a critical determinant of adherence and psychological wellbeing in cancer survivors. Qualitative studies indicate that group dynamics foster a sense of camaraderie and mutual understanding that is difficult to achieve in individual exercise, acting as a key driver for long-term participation [37, 38]. Moreover, structured group exercise models not only facilitate peer support [39] but, when combined with supervision by qualified professionals, create a safe environment that enhances patient self-efficacy and reduces fear of movement [33, 35, 37].

The findings of this study suggest several concrete strategies to enhance exercise participation among cancer patients. Embedding qualified exercise professionals within oncology teams can provide tailored guidance, monitor safety, and offer ongoing support, directly increasing adherence. In the same line, implementing standardized referral pathways can streamline the identification and enrollment of patients who would benefit from structured exercise programs [13, 40]. Complementing this with patient education frameworks can address knowledge gaps, clarify the benefits of physical activity, and align recommendations with patient preferences, thereby translating positive attitudes into actual engagement.

Using the MOVE-Onco to assess individual barriers, motivators, and contextual constraints enables clinicians to design personalized exercise prescriptions that consider both physical limitations and psychosocial factors. Its longitudinal application allows for monitoring changes over time, informing adaptive interventions during prehabilitation, rehabilitation, or survivorship programs. Additionally, incorporating socially supported exercise opportunities, such as group sessions or peer-based programs, can enhance motivation, foster engagement, and reduce dropout. Collectively, these approaches provide a practical roadmap for bridging the gap between patient motivation and effective, sustained exercise behavior in oncology care.

This study has several notable strengths. Its rigorous and transparent development process, combined with the multidisciplinary involvement of experts, ensured that the MOVE-Onco is both patient-centered and culturally adapted. The high internal consistency observed for the barriers and facilitators sections further supports the reliability of these domains. However, several limitations should be acknowledged. The preliminary sample was relatively small, geographically restricted to Spain, and predominantly composed of women with a specific type of cancer who were already physically active, which may limit the generalizability of the findings. Expert participation was also confined to a single country. Importantly, the present study provides only preliminary evidence of the psychometric properties of the MOVE-Onco questionnaire. While internal consistency was satisfactory, other key aspects of validation—such as factor structure, construct validity, and test–retest reliability—were not assessed and require further investigation in larger samples. In addition, the study did not directly assess socioeconomic status, income, or geographic accessibility variables; therefore, conclusions regarding social gradients in exercise access remain exploratory. Future research should focus on comprehensive psychometric validation of the MOVE-Onco in larger and more diverse populations, incorporating exploratory and confirmatory factor analyses, test–retest reliability, and assessments of convergent and discriminant validity. Cross-cultural adaptations for other Spanish-speaking contexts, as well as translations into additional languages, are also warranted. Finally, future work should develop shorter, clinically feasible versions of MOVE-Onco and incorporate explicit socioeconomic and territorial indicators to better characterize equity-related disparities and facilitate implementation in routine oncology care.

In conclusion, the MOVE-Onco questionnaire provides a patient-centered tool to identify informational and structural barriers to exercise in oncology settings. Preliminary findings suggest that limited professional guidance and low awareness of available resources may be relevant barriers to exercise participation in this context. However, these results should be interpreted as exploratory and hypothesis-generating, and further research is needed to confirm the role of structural factors in exercise oncology care.

## Supplementary Information

Below is the link to the electronic supplementary material.

Supplementary file1 (PDF 76 KB)

Supplementary file2 (PDF 181 KB)

Supplementary file3 (PDF 139 KB)

Supplementary file4 (PDF 104 KB)

## Data Availability

The data underlying this article will be shared at reasonable request to the corresponding authors.
