# Supplementary material for: Informational and structural barriers to exercise oncology care in Spain: development of the MOVE-Onco questionnaire and preliminary findings
Source: Clin Transl Oncol. 2026 May 18;28(10):4958–71. doi: 10.1007/s12094-026-04375-9 (PMC13601178; doi:10.1007/s12094-026-04375-9)
Supplement: Supplementary file 1 [file 12094_2026_4375_MOESM1_ESM.pdf]

Supplementary Material 1. Open-ended questions.

¿Le resulta posible sacar tiempo de su rutina diaria para un programa de ejercicio?

¿Está interesado en formar parte de un programa de ejercicio?

¿En qué tipo de ejercicios físicos estaría más interesado en formar parte?

¿Cuánto tiempo crees que podrías realizar ejercicio físico de manera continua?

¿En qué momento del día preferirías realizar ejercicio físico?

¿Dónde preferirías que se realizase el programa de ejercicio?

¿Con quién te gustaría realizar ejercicio físico?

¿Quién te gustaría que dirija el programa de ejercicio físico?

¿Cuándo prefieres o hubieras preferido comenzar un programa de entrenamiento?

¿Cómo te gustaría recibir información sobre los programas de ejercicio físico disponibles?

¿Cuáles crees que podrían ser los efectos adversos del ejercicio físico?

¿Cuáles crees que son los posibles beneficios del ejercicio físico regular?

¿Algún profesional sanitario te ha enseñado ejercicios para realizar después del tratamiento contra el cáncer?

¿Cuándo crees que debería comenzar el programa de ejercicio físico?

¿Crees que el ejercicio físico puede mejorar tu condición?

¿Sabes de algún hospital o centro cercano donde se realicen programas de ejercicio físico oncológico?

¿Por qué no te apuntas a un programa de ejercicio físico?

Incluso después de conocer los beneficios del ejercicio y las recomendaciones de un profesional de la salud, ¿qué le impide hacer ejercicio?

¿Qué aspectos hacen que no puedas acceder fácilmente a los servicios de ejercicio físico?

¿Qué factores físicos cree que reducen su participación en el ejercicio físico?

Complete la frase: Me sentiría motivado para realizar ejercicio si...

¿Qué persona le motiva a realizar ejercicio físico?

Complete la frase: Me sentiría motivado si esta persona me acompaña al lugar de ejercicio físico...

Complete la frase: Me gustaría participar en un programa de ejercicio físico si...

¿Qué le haría practicar ejercicio físico regularmente?
