# Supplementary material for: Informational and structural barriers to exercise oncology care in Spain: development of the MOVE-Onco questionnaire and preliminary findings
Source: Clin Transl Oncol. 2026 May 18;28(10):4958–71. doi: 10.1007/s12094-026-04375-9 (PMC13601178; doi:10.1007/s12094-026-04375-9)
Supplement: Supplementary file 3 [file 12094_2026_4375_MOESM3_ESM.pdf]

Supplementary Material 3. Agreement percentages in Delphi process.

**Round 1 questionnaire with percentage consensus. Consensus was defined as a priori as equal to or greater than 75%**		**Round 2 questionnaire with percentage consensus. Consensus defined a priori as equal to or greater than 75%**	
**Question**	**Percent consensus**	**Question**	**Percent Consensus**
**SECCIÓN 1/5: GENERAL**			
¿Realizas, actualmente, ejercicio físico de forma regular?<br>Si.<br>No.	100%	¿Realizas, actualmente, ejercicio físico de forma regular?<br>Si.<br>No.	100%
¿Realizabas ejercicio físico antes del diagnóstico de cáncer=<br>Sí.<br>No.<br>Si has contestado que sí, ¿cuántos días a la semana y qué duración por cada uno de esos días? ____________	100%	¿Realizabas ejercicio físico antes del diagnóstico de cáncer?<br>Sí.<br>No.<br>Si has contestado que sí, ¿cuántos días a la semana y qué duración por cada uno de esos días? ______ días, ______ minutos.	100%
¿Con qué frecuencia semanal te gustaría hacer ejercicio físico si tu salud lo permitiera?<br>1–2 días<br>3–4 días<br>5 o más días<br>No lo sé	68.8%	Deleted	
¿Cómo te desplazas al médico?<br>En vehículo propio.<br>En transporte público.<br>En taxi.<br>Caminando.<br>En bicicleta.	93.7%	¿Cómo te desplazas al médico?<br>En vehículo propio.<br>En transporte público.<br>En taxi.<br>Caminando.<br>En bicicleta.	100%
¿Quién te cuida cuando estás enfermo?<br>Pareja.<br>Hijos.<br>Otro miembro de tu familia.<br>Cuidador/a<br>Nadie.	87.5%	¿Quién te cuida cuando estás enfermo/a?<br>Pareja.<br>Hijos.<br>Otro miembro de tu familia.<br>Asistente personal/ cuidador de ayuda a domicilio.<br>Nadie.	92.3%
¿Cómo de difícil es viajar para ti?<br>Muy difícil.<br>Difícil, pero puedo afrontarlo.<br>No tengo dificultad.	87.5%	¿Cómo de difícil es viajar para ti?<br>Muy difícil.<br>Difícil, pero puedo afrontarlo.<br>No tengo dificultad.	92.3%
**SECCIÓN 2/5: PREFERENCIAS**			
¿Te resulta posible sacar tiempo de tu rutina diaria	100%	¿Te resulta posible sacar tiempo de tu rutina diaria	100%

para realizar un programa de ejercicio?Sí.<br>No.<br>A veces.		para realizar un programa de ejercicio?<br>Sí.<br>No.<br>A veces.	
¿Estás interesado/a en participar en un programa de ejercicio físico?<br>Si.<br>No.<br>No estoy seguro/a.	100%	¿Estás interesado/a en participar en un programa de ejercicio físico?<br>Sí.<br>No.<br>No estoy seguro/a	100%
¿En qué tipo de ejercicios físico estarías más interesado de participar?<br>Caminar.<br>Montar en bicicleta.<br>Correr/trotar.<br>Bailar.<br>Yoga/Pilates.<br>Ejercicios con pesas.<br>Ejercicios para mejorar la flexibilidad.<br>Nadar.<br>Entrenamiento guiado.<br>Cinta de correr.<br>Deportes individuales (pádel, tenis, golf, etc.).<br>Deportes colectivos (fútbol, baloncesto, rugby, hockey, voleibol).<br>Montar a caballo.<br>Patinaje.<br>Ningún ejercicio.	100%	¿En qué tipos de ejercicios físico estarías más interesado/a en participar? Puedes seleccionar varios.<br>Caminar.<br>Montar en bicicleta.<br>Correr/trotar.<br>Bailar.<br>Yoga/Pilates.<br>Ejercicios con pesas.<br>Ejercicios para mejorar la flexibilidad.<br>Nadar.<br>Entrenamiento guiado.<br>Cinta de correr.<br>Deportes individuales (pádel, tenis, golf, etc.).<br>Deportes colectivos (fútbol, baloncesto, rugby, hockey, voleibol).<br>Montar a caballo.<br>Patinaje.<br>Ningún ejercicio.	100%
¿Cuánto tiempo crees que podrías realizar ejercicio físico de manera continua?<br>Menos de 10 min.<br>Entre 10 y 20 min.<br>Entre 20 y 30 min.<br>Más de 30 min.	93.7%	¿Cuánto tiempo crees que podrías realizar ejercicio físico de manera continua?<br>Menos de 10 min.<br>Entre 10 y 20 min.<br>Entre 20 y 30 min.<br>Más de 30 min.	100%
¿Preferirías ejercicios de intensidad baja, moderada o alta?<br>Baja (caminar, estiramientos suaves)<br>Moderada (bicicleta, nadar, yoga)<br>Alta (correr, deportes de equipo)<br>Depende del día	68.5%	Deleted	
10. ¿En qué momento del día preferirías realizar ejercicio físico?<br>Mañana.	100%	¿En qué momento del día preferirías realizar ejercicio físico?<br>Mañana.	

A media tarde.<br>Tarde/noche.<br>No tengo preferencia.		A media tarde.<br>Tarde/noche.<br>No tengo preferencia	
¿Dónde preferirías que se realizase el programa de ejercicio?<br>En casa.<br>En el gimnasio.<br>En un parque.<br>En alguna asociación.<br>En un centro multidisciplinar.<br>En el hospital/centro de salud.<br>Otros (especifica ______).	100%	¿Dónde preferirías que se realizase el programa de ejercicio?<br>En casa.<br>En el gimnasio.<br>En un parque.<br>En alguna asociación.<br>En un centro multidisciplinar.<br>En el hospital/centro de salud.<br>Otros (especifica: _____).	100%
¿Con quién te gustaría realizar ejercicio físico?<br>Solo, siguiendo las instrucciones de un entrenador personal.<br>Con otros supervivientes de cáncer.<br>Personas sin cáncer.<br>Familiares.<br>Amigos.<br>Otros (especifica _______)<br>Si no has respondido la b, especifica la razón ____________________	100%	¿Con quién te gustaría realizar ejercicio físico?<br>Solo/a, siguiendo las instrucciones de un entrenador personal.<br>Con otros supervivientes de cáncer.<br>Público general.<br>Familiares.<br>Amigos.<br>Otros (especifica: ______).<br>Si no has respondido la b), especifica la razón: ______________________.	100%
¿Quién te gustaría que dirija el programa de ejercicio físico?<br>Fisioterapeuta.<br>Oncólogo.<br>Entrenador personal (educador físico-deportivo).<br>Internet<br>Otro, especifica _________	81.25%	¿Quién te gustaría que dirija el programa de ejercicio físico?<br>Fisioterapeuta.<br>Oncólogo.<br>Entrenador personal (educador físico-deportivo).<br>Yo mismo, con información extraída de Internet.<br>Yo mismo, con información extraída de libros y guías sobre ejercicio físico.<br>Otro (especifica: ___).	92.3%
¿Cuándo prefieres o hubieras preferido comenzar un programa de entrenamiento?<br>Antes del tratamiento.<br>Durante el tratamiento.<br>De 0 a 6 meses después de completar el tratamiento.<br>De 7 a 12 meses después de completar el tratamiento.<br>Un año o más después de completar el tratamiento.	87.5%	¿Cuándo prefieres o hubieras preferido comenzar un programa de entrenamiento?<br>Antes del tratamiento.<br>Durante el tratamiento.<br>0 a 6 meses después de completar el tratamiento.<br>De 7 a 12 meses después de completar el tratamiento.<br>Un año o más después de completar el tratamiento.	100%

¿Cómo te gustaría recibir información sobre los programas de ejercicio físico disponibles?<br>Teléfono.<br>Folleto.<br>Videos.<br>Charlas o talleres presenciales con un experto.<br>Aplicación móvil con ejercicios.<br>Otros (especifica_____)	100%	¿Cómo te gustaría recibir información sobre los programas de ejercicio físico disponibles?<br>Teléfono.<br>Folleto.<br>Videos.<br>Charlas o talleres presenciales con un experto.<br>Aplicación móvil con ejercicios.<br>Otros (especifica: _____).	92.3%
**SECCIÓN 3/5: CONOCIMIENTO**			
¿Cuáles crees que podrían ser los efectos adversos del ejercicio físico?<br>Lesión.<br>Deterioro de la masa muscular. Tiempo de recuperación más prolongado debido al dolor inducido por el ejercicio.<br>El ejercicio no tiene efectos adversos.<br>Dolor.<br>Fatiga.<br>Otros (especifica _____)	100%	¿Cuáles crees que podrían ser los efectos adversos del ejercicio físico?<br>Lesión.<br>Deterioro de la masa muscular.<br>Tiempo de recuperación más prolongado debido al dolor inducido por el ejercicio.<br>El ejercicio no tiene efectos adversos.<br>Dolor.<br>Fatiga.<br>Otros (especifica: ______).	92.3%
¿Cuáles crees que son los posibles beneficios del ejercicio físico regular?<br>Mejora la fuerza muscular.<br>Ayuda a reducir la fatiga.<br>Mantiene o mejora la salud.<br>Reduce el dolor.<br>Previene las caídas.<br>Reduce la ansiedad.<br>Reduce los niveles de depresión.<br>Reduce el tiempo de reincorporación laboral.<br>Permite ser más activo a nivel social.<br>Ninguno de los anteriores.<br>Todos los anteriores.<br>Otros (especifica______)	100%	¿Cuáles crees que son los posibles beneficios del ejercicio físico regular?<br>Mejora la fuerza muscular.<br>Ayuda a reducir la fatiga.<br>Mantiene o mejora la salud.<br>Reduce el dolor.<br>Previene las caídas.<br>Reduce la ansiedad.<br>Reduce los niveles de depresión.<br>Reduce el tiempo de reincorporación laboral.<br>Permite ser más activo a nivel social.<br>Ninguno de los anteriores.<br>Todos los anteriores.<br>Otros (especifica:______).	92.3%
¿Algún profesional sanitario te ha enseñado ejercicios para realizar después del tratamiento contra el cáncer?<br>Si.<br>NoSi, pero no recuerdo los ejercicios.	100%	¿Algún profesional sanitario te ha enseñado ejercicios para realizar después del tratamiento contra el cáncer?<br>Sí.<br>No.<br>Sí, pero no recuerdo los ejercicios.	100%

¿Cuándo crees que debería comenzar el programa de ejercicio físico?<br>Durante el tratamiento.<br>En los 3 meses posteriores a la finalización del tratamiento.<br>Entre 3-6 meses posteriores a la finalización del tratamiento.<br>Entre 7-12 meses posteriores a la finalización del tratamiento.<br>Un año o más después de la finalización del tratamiento.	87.5%	¿Cuándo crees que debería comenzar el programa de ejercicio físico?<br>Durante el tratamiento.<br>En los 3 meses posteriores a la finalización del tratamiento.<br>Entre 3-6 meses posteriores a la finalización del tratamiento.<br>Entre 7-12 meses posteriores a la finalización del tratamiento.<br>Un año o más después de la finalización del tratamiento.	92.3%
¿Crees que el ejercicio físico puede mejorar tu condición? Sí.<br>No.<br>Quizás.	100%	¿Crees que el ejercicio físico puede mejorar tu condición?<br>Sí.<br>No.<br>Quizás.	100%
¿Sabes de algún hospital o centro cercano donde se realicen programas de ejercicio físico oncológico?<br>a. Sí.<br>b. No.	100%	¿Sabes de algún hospital o centro cercano donde se realicen programas de ejercicio físico oncológico?<br>Sí.<br>No.	92.3%
**SECCIÓN 4/5: BARRERAS**			
A continuación, encontrarás una serie de afirmaciones sobre las razones por las que no participas en un programa de ejercicio físico. Por favor, indica en qué medida estás de acuerdo con cada afirmación utilizando una escala del 1 al 5, donde:<br>1 = Completamente en desacuerdo<br>5 = Completamente de acuerdo<br>Me siento demasiado cansado/a para hacer ejercicio.<br>No tengo suficiente fuerza para hacer ejercicio. No creo que el ejercicio marque una diferencia significativa en mi salud. d. El dolor me impide hacer ejercicio.<br>Tengo miedo a caerme.	100%	A continuación, encontrarás una serie de afirmaciones sobre las razones por las que no participas en un programa de ejercicio físico. Por favor, indica en qué medida estás de acuerdo con cada afirmación utilizando una escala del 1 al 5, donde: 1 = Completamente en desacuerdo; 5 = Completamente de acuerdo.<br><br>Me siento demasiado cansado/a para hacer ejercicio.<br>No tengo suficiente fuerza para hacer ejercicio. No creo que el ejercicio marque una diferencia significativa en mi salud. El dolor me impide hacer ejercicio.	100%

Siento dificultad para respirar.<br>No me han informado los profesionales sanitarios.<br>No tengo tiempo.<br>No tengo motivación o ganas.<br>No puedo permitírmelo económicamente.<br>No conozco sitios especializados en ejercicio físico oncológico.<br>Siento falta de apoyo.<br>Siento vergüenza.<br>Otros (especificar ______).		Tengo miedo a caerme.<br>Siento dificultad para respirar.<br>Los profesionales no me han informado acerca de cómo debo realizar correctamente ejercicio físico con mi diagnóstico<br>No tengo tiempo.<br>No tengo motivación o ganas.<br>No puedo permitírmelo económicamente.<br>No conozco sitios especializados en ejercicio físico oncológico.<br>Siento falta de apoyo en mi entorno.<br>Siento vergüenza.<br>Tengo responsabilidades domésticas que me lo impiden.<br>Otros (especificar______).	
Incluso conociendo los beneficios del ejercicio físico y habiéndotelo recomendado un profesional sanitario, indica en qué medida estás de acuerdo con cada una de estas afirmaciones, usando la misma escala del 1 al 5.<br><br>No consigo empezar a hacer ejercicio.<br>No soy capaz de mantener una rutina de ejercicio.<br>El precio de los servicios de ejercicio físico es elevado.<br>El progreso en la efectividad del tratamiento es lento.<br>No me siento seguro para salir y hacer ejercicio.<br>Otros factores (especificar __________) me impiden hacer ejercicio.	93.5%	Incluso conociendo los beneficios del ejercicio físico y habiéndotelo recomendado un profesional sanitario, indica en qué medida estás de acuerdo con cada una de estas afirmaciones, utilizando una escala del 1 al 5<br><br>No consigo empezar a hacer ejercicio.<br>No soy capaz de mantener una rutina de ejercicio.<br>El precio de los servicios de ejercicio físico es elevado.<br>El progreso en la efectividad del tratamiento es lento.<br>No me siento seguro para salir y hacer ejercicio.<br>Otros factores (especificar ___________) me impiden hacer ejercicio.	100%
¿Puede acceder fácilmente a los servicios de ejercicio físico?<br>Sí.<br>No.	62.5%	Deleted	
A continuación, encontrarás afirmaciones sobre los factores que pueden afectar tu participación en un	100%	A continuación, se presentan afirmaciones sobre el acceso a servicios de ejercicio físico. Indica en qué	100%

programa de ejercicio físico. Utiliza la escala del 1 al 5 para indicar tu nivel de acuerdo.<br><br>Mi sobrepeso reduce mi participación en el ejercicio físico.<br>El dolor reduce mi participación en el ejercicio físico.<br>La inflamación o el linfedema me impiden realizar ejercicio físico.<br>La rigidez articular reduce mi participación en el ejercicio físico.<br>Tengo problemas para retener la orina y eso reduce mi participación en un programa de ejercicio físico.<br>Nadie me apoya para realizar ejercicio físico.<br>Siento vergüenza al realizar ejercicio físico.<br>No tengo ánimo para realizar ejercicio físico.<br>Otros factores (especificar cuál/es_______________) afectan mi participación en el ejercicio físico.		medida estás de acuerdo con cada una de estas afirmaciones, utilizando una escala del 1 al 5, donde: 1 = Completamente en desacuerdo; 5 = Completamente de acuerdo.<br>Mi sobrepeso reduce mi participación en el ejercicio físico.<br>El dolor reduce mi participación en el ejercicio físico.<br>La inflamación o el linfedema (inflamación de las extremidades superiores y/o inferiores) me impiden realizar ejercicio físico.<br>La rigidez articular reduce mi participación en el ejercicio físico.<br>Tengo problemas para retener la orina y eso reduce mi participación en un programa de ejercicio físico.<br>Nadie me apoya para realizar ejercicio físico.<br>Siento vergüenza al realizar ejercicio físico.<br>No tengo ánimo para realizar ejercicio físico.<br>Tengo responsabilidades familiares que reduce mi participación.<br>Otros factores (especificar cuál/es_____) afectan mi participación en el ejercicio físico.	
**SECCIÓN 5/5: FACILITADORES**			
A continuación, encontrarás afirmaciones sobre lo que te motivaría a hacer ejercicio físico. Indica tu nivel de acuerdo con cada una en una escala del 1 al 5.<br>Me motivaría a hacer ejercicio si me ayudase a mantener el equilibrio, la fuerza y la agudeza mental.<br>Me motivaría a hacer ejercicio si me sintiera renovado/a después del ejercicio.<br>Me motivaría a hacer ejercicio si previniera el	100%	A continuación, encontrarás afirmaciones sobre lo que te motivaría a hacer ejercicio físico. Indica en qué medida estás de acuerdo con cada afirmación utilizando una escala del 1 al 5, donde: 1 = Completamente en desacuerdo; 5 = Completamente de acuerdo.<br><br>Me motivaría a hacer ejercicio si me ayudase a mantener el equilibrio, la fuerza y la agudeza mental.	100%

deterioro de mi estado actual.<br>Me motivaría a hacer ejercicio si mejorara mi calidad de vida.<br>Me motivaría a hacer ejercicio si conociera previamente los beneficios.<br>Otros factores (especificar cuáles______) me motivarían a hacer ejercicio físico.		Me motivaría a hacer ejercicio si me sintiera renovado/a después del ejercicio.<br>Me motivaría a hacer ejercicio si previniera el deterioro de mi estado actual.<br>Me motivaría a hacer ejercicio si mejorara mi calidad de vida.<br>Me motivaría a hacer ejercicio si conociera previamente los beneficios.<br>Otros factores (especificar cuáles______) me motivarían a hacer ejercicio físico.	
A continuación, encontrarás una serie de afirmaciones sobre las personas que te motivan a realizar ejercicio físico. Por favor, indica en qué medida estás de acuerdo con cada afirmación utilizando una escala del 1 al 5.<br>Mi pareja me motiva a participar.<br>Mis hermanos/as me motivan a participar.<br>Mis hijos/as me motivan a participar.<br>Mis amigos/as me motivan a participar.<br>Mis familiares me motivan a participar.<br>Nadie me motiva a participar.<br>Otros (especificar ______) me animan a participar.	100%	A continuación, encontrarás una serie de afirmaciones sobre las personas que te motivan a realizar ejercicio físico. Indica en qué medida estás de acuerdo con cada afirmación utilizando una escala del 1 al 5, donde: 1 = Completamente en desacuerdo; 5 = Completamente de acuerdo.<br>Mi pareja me motiva a participar.<br>Mis hermanos/as me motivan a participar.<br>Mis hijos/as me motivan a participar.<br>Mis amigos/as me motivan a participar.<br>Mis familiares me motivan a participar.<br>Nadie me motiva a participar.<br>Otros (especificar _______) me animan a participar.	100%
A continuación, encontrarás afirmaciones sobre tu motivación para hacer ejercicio si una persona específica te acompañara. Indica tu nivel de acuerdo con cada una en una escala del 1 al 5.<br><br>Me sentiría motivado/a si mi pareja me acompaña.	93.7%	A continuación, encontrarás afirmaciones sobre tu motivación para hacer ejercicio si una persona específica te acompañara. Indica en qué medida estás de acuerdo con cada afirmación utilizando una escala del 1 al 5, donde: 1 = Completamente en desacuerdo; 5 =	100%

Me sentiría motivado/a si mi hermanos/as me acompaña.<br>Me sentiría motivado/a si mi hijo/s me acompaña.<br>Me sentiría motivado/a si un amigo/a me acompaña.<br>Me sentiría motivado/a si algún familiar me acompaña.<br>Me sentiría motivado/a si nadie me acompaña.<br>Me sentiría motivado/a si mi mascota me acompaña.<br>Me sentiría motivado/a si (especificar __________) me acompaña.		Completamente de acuerdo.<br>Me sentiría motivado/a si mi pareja me acompaña.<br>Me sentiría motivado/a si mi hermanos/as me acompaña.<br>Me sentiría motivado/a si mi hijo/s me acompaña.<br>Me sentiría motivado/a si un amigo/a me acompaña.<br>Me sentiría motivado/a si algún familiar me acompaña.<br>Me sentiría motivado/a si nadie me acompaña.<br>Me sentiría motivado/a si mi mascota me acompaña.<br>Me sentiría motivado/a si (especificar ___________) me acompaña.	
A continuación, se presentan afirmaciones sobre las condiciones que te harían más propenso/a a participar en un programa de ejercicio físico. Indica tu nivel de acuerdo con cada una en una escala del 1 al 5.<br>Participaría en un programa de ejercicio si fuera de bajo coste o gratis.<br>Participaría en un programa de ejercicio si el centro estuviera cerca.<br>Participaría en un programa de ejercicio si alguien me acompañara.<br>Participaría en un programa de ejercicio si fuera accesible a nivel de transporte (bien comunicado, parking, paradas de autobús, etc.).<br>Participaría en un programa de ejercicio si las sesiones fueran en grupo con otros pacientes con cáncer.<br>Participaría en un programa de ejercicio si el horario del programa fuese flexible.<br>Otros (especificar ______).	100%	A continuación, se presentan afirmaciones sobre las condiciones que te harían más propenso/a a participar en un programa de ejercicio físico. Indica en qué medida estás de acuerdo con cada afirmación utilizando una escala del 1 al 5, donde: 1 = Completamente en desacuerdo; 5 = Completamente de acuerdo.<br><br>Participaría en un programa de ejercicio si fuera de bajo coste o gratis.<br>Participaría en un programa de ejercicio si el centro estuviera cerca.<br>Participaría en un programa de ejercicio si alguien me acompañara.<br>Participaría en un programa de ejercicio si fuera accesible a nivel de transporte (bien comunicado, parking, paradas de autobús, etc.).<br>Participaría en un programa de ejercicio si las sesiones fueran en grupo con otros pacientes con cáncer.<br>Participaría en un programa de ejercicio si el	100%

		horario del programa fuese flexible.<br>Otros (especificar ______).	
A continuación, encontrarás afirmaciones sobre lo que te motivaría a hacer ejercicio de manera regular. Indica tu nivel de acuerdo en una escala del 1 al 5.<br><br>Practicaría ejercicio físico regularmente si disfrutara al realizarlo.<br>Practicaría ejercicio físico regularmente si mi familia me motivara a ello.<br>Practicaría ejercicio físico regularmente si me previniera de recaer.<br>Practicaría ejercicio físico regularmente si sintiera que tengo el control sobre mi salud.<br>Practicaría ejercicio físico regularmente si redujese los efectos secundarios del tratamiento.<br>Otros factores (especificar _______) me motivarían a practicar ejercicio físico regularmente.	100%	A continuación, encontrarás afirmaciones sobre lo que te motivaría a hacer ejercicio de manera regular. Indica en qué medida estás de acuerdo con cada afirmación utilizando una escala del 1 al 5, donde: 1 = Completamente en desacuerdo; 5 = Completamente de acuerdo.<br><br>Practicaría ejercicio físico regularmente si disfrutara al realizarlo.<br>Practicaría ejercicio físico regularmente si mi familia me motivara a ello.<br>Practicaría ejercicio físico regularmente si me previniera de recaer.<br>Practicaría ejercicio físico regularmente si sintiera que tengo el control sobre mi salud.<br>Practicaría ejercicio físico regularmente si redujese los efectos secundarios del tratamiento.<br>Otros factores (especificar _______) me motivarían a practicar ejercicio físico regularmente.	100%
