# Supplementary material for: Informational and structural barriers to exercise oncology care in Spain: development of the MOVE-Onco questionnaire and preliminary findings
Source: Clin Transl Oncol. 2026 May 18;28(10):4958–71. doi: 10.1007/s12094-026-04375-9 (PMC13601178; doi:10.1007/s12094-026-04375-9)
Supplement: Supplementary file 4 [file 12094_2026_4375_MOESM4_ESM.pdf]

Supplementary Material 4. Cover letter with the Delphi methodology and instructions.

Motivators, Obstacles, Values, and Exercise in Oncology (MOVE-Onco): PROCESO DELPHI RONDA 1.

Estimadas/os profesionales,

Desde el equipo investigador de la **Cátedra PROMETEO: Promoción del ejercicio físico en el paciente oncológico**, financiada por la Asociación Española contra el Cáncer, queremos invitarles a participar en un proyecto clave para mejorar la calidad asistencial de los pacientes oncológicos.

¿Por qué es importante su colaboración?

Estamos analizando los conocimientos, barreras y facilitadores relacionados con el ejercicio físico en pacientes con cáncer. Esta información permitirá diseñar estrategias que refuercen el papel del ejercicio físico en el tratamiento integral de los pacientes.

En este marco, hemos desarrollado el cuestionario **Motivators, Obstacles, Values, and Exercise in Oncology (MOVE-Onco)**. Su participación es esencial para que refleje fielmente la visión de los profesionales en España. Por ello, le invitamos a formar parte de la primera fase de nuestro estudio.

¿En qué consiste su participación?

Se utilizará el **Método Delphi**, un proceso anónimo y reiterativo que permite a expertos aportar opiniones hasta alcanzar consenso.
En este estudio se realizarán **dos rondas**, ambas mediante formularios de Google Forms.

Deberá completar la siguiente encuesta, donde se solicita:

1. Valorar la relevancia de cada ítem (Sí/No).
2. Indicar su claridad (Sí/No).
3. Proponer modificaciones en la redacción de los ítems o sus respuestas, si lo considera necesario.

- FECHA LÍMITE: El proceso se repetirá en **dos rondas**, la **Ronda I el** plazo hasta el **8 de enero**.

Confidencialidad

Todas las respuestas serán tratadas con absoluta confidencialidad y se emplearán únicamente con fines de investigación.

Agradecemos de antemano su tiempo y compromiso con este proyecto.

Atentamente,
Equipo de investigación de la Cátedra PROMETEO

Motivators, Obstacles, Values, and Exercise in Oncology (MOVE-Onco): PROCESO DELPHI RONDA 2

Estimadas/os profesionales,

Desde el equipo investigador de la **Cátedra PROMETEO: Promoción del ejercicio físico en el paciente oncológico**, financiada por la **Asociación Española contra el Cáncer**, queremos agradecer su valiosa participación en la primera ronda del estudio y convocarles a la **Ronda 2** del proceso Delphi.

¿En qué consiste su participación?

Deberá completar un nuevo formulario (Google Forms), en el que se solicita:

1. Valorar el **grado de relevancia** de los ítems revisados (escala Likert)

2. Aportar comentarios adicionales o sugerencias, si lo considera oportuno.

    1. FECHA LÍMITE: hasta el **31 de enero.**

Confidencialidad

Todas las respuestas serán tratadas con absoluta confidencialidad y se emplearán únicamente con fines de investigación.

Agradecemos de antemano su tiempo y compromiso con este proyecto.

Atentamente,
Equipo de investigación de la Cátedra PROMETEO
